# Pericytes and vascular smooth muscle cells in central nervous system arteriovenous malformations

**DOI:** 10.3389/fphys.2023.1210563

**Published:** 2023-08-04

**Authors:** Sera Nakisli, Alfonso Lagares, Corinne M. Nielsen, Henar Cuervo

**Affiliations:** ^1^ Department of Biological Sciences, Ohio University, Athens, OH, United States; ^2^ Neuroscience Program, Ohio University, Athens, OH, United States; ^3^ Department of Neurosurgery, University Hospital 12 de Octubre, Madrid, Spain; ^4^ Department of Surgery, Universidad Complutense de Madrid, Madrid, Spain; ^5^ Instituto de Investigación Imas12, Madrid, Spain; ^6^ Molecular and Cellular Biology Program, Ohio University, Athens, OH, United States; ^7^ Centro Nacional de Investigaciones Cardiovasculares Carlos III (F.S.P), Madrid, Spain

**Keywords:** arteriovenous malformation, central nervous system, mural cell, pericyte, smooth muscle cell, vascular malformations, brain vessels

## Abstract

Previously considered passive support cells, mural cells—pericytes and vascular smooth muscle cells—have started to garner more attention in disease research, as more subclassifications, based on morphology, gene expression, and function, have been discovered. Central nervous system (CNS) arteriovenous malformations (AVMs) represent a neurovascular disorder in which mural cells have been shown to be affected, both in animal models and in human patients. To study consequences to mural cells in the context of AVMs, various animal models have been developed to mimic and predict human AVM pathologies. A key takeaway from recently published work is that AVMs and mural cells are heterogeneous in their molecular, cellular, and functional characteristics. In this review, we summarize the observed perturbations to mural cells in human CNS AVM samples and CNS AVM animal models, and we discuss various potential mechanisms relating mural cell pathologies to AVMs.

## Arteriovenous malformations

Arteriovenous malformations (AVMs) are vascular anomalies that are characterized by having a widened arteriovenous (AV) connection. This increased AV diameter permits increased blood flow and pressure, leading to microhemorrhages, poor nutrient-waste exchange, and tortuous vessels, which may form into a vessel entanglement called a nidus (Nielsen et al., 2016; Varshneya et al., 2019). Clinically, AVMs can develop in many tissues throughout the body, though most develop as focal lesions in patients. In fact, patients that present with multi-tissue AVMs usually have an inherited syndrome, such as Hereditary Hemorrhagic Telangiectasia (HHT), discussed in more detail below, in which AVMs can be observed in nose, lung, brain, and liver (Kim et al., 2018; Snodgrass et al., 2021). Consequences of AVMs depend on the severity of the lesion and on the tissue affected. For example, AVMs in the human CNS can impair neurological function and greatly impact quality of life. While CNS AVMs develop primarily in the brain, AVMs can also form in the retina and spinal cord (Vieira et al., 2020). The etiologies of these distinct CNS AVMs are discussed below.

### Brain AVMs

**FIGURE 1:**
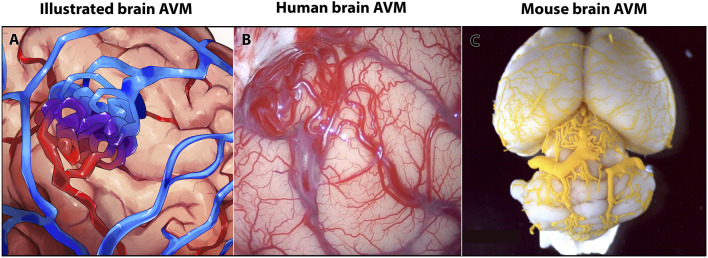
Human and mouse brain arteriovenous malformations (AVMs) show similar anatomy of vascular lesions. **(A)** Schematic of a brain AVM. **(B)** Image of AVM nidus on the surface of a human brain, occipital cortex. **(C)** Image of MICROFIL^®^ vascular cast of postnatal day 35 mouse brain AVM.

Brain AVMs account for approximately 2% of all hemorrhagic strokes but cause almost half of all hemorrhagic strokes in children and young adults (Mahmoud et al., 2010; Nielsen et al., 2016; Solomon and Connolly, 2017). A hallmark of brain AVMs is the direct delivery of blood from artery to vein via an AV shunt, which forms in place of healthy capillary AV connections (Nielsen et al., 2016; Adhicary et al., 2022). In humans, brain AVMs can present as a tangle of enlarged, tortuous vessels—clinically termed a nidus (Figures 1A, B)—that may place contact pressure on surrounding brain tissue. The shunt effect driven by the AVM can cause hypoperfusion of surrounding brain tissue (steal effect). Additionally, because of the altered brain perfusion and increased pressure in both in the nidus and in AVM veins, brain AVMs have a risk of rupture that is around 1% per year. On average, the risk of spontaneous rupture and hemorrhage is low; however, there are factors that can increase this risk, such as older patient age, deep AVM location, and deep draining venous system (Stapf et al., 2006; Kim et al., 2014). Additionally, risk of hemorrhage is significantly increased in AVMs with previous hemorrhagic presentation (Gross and Du, 2013). By contrast, AVM size was not associated with increased rupture risk (Kim et al., 2014). Patients presenting with more than one risk factor have increased likelihood of rupture; patients with all three risk factors can have up to 34% chance of incurring a brain hemorrhage (Stapf et al., 2006).

**FIGURE 2:**
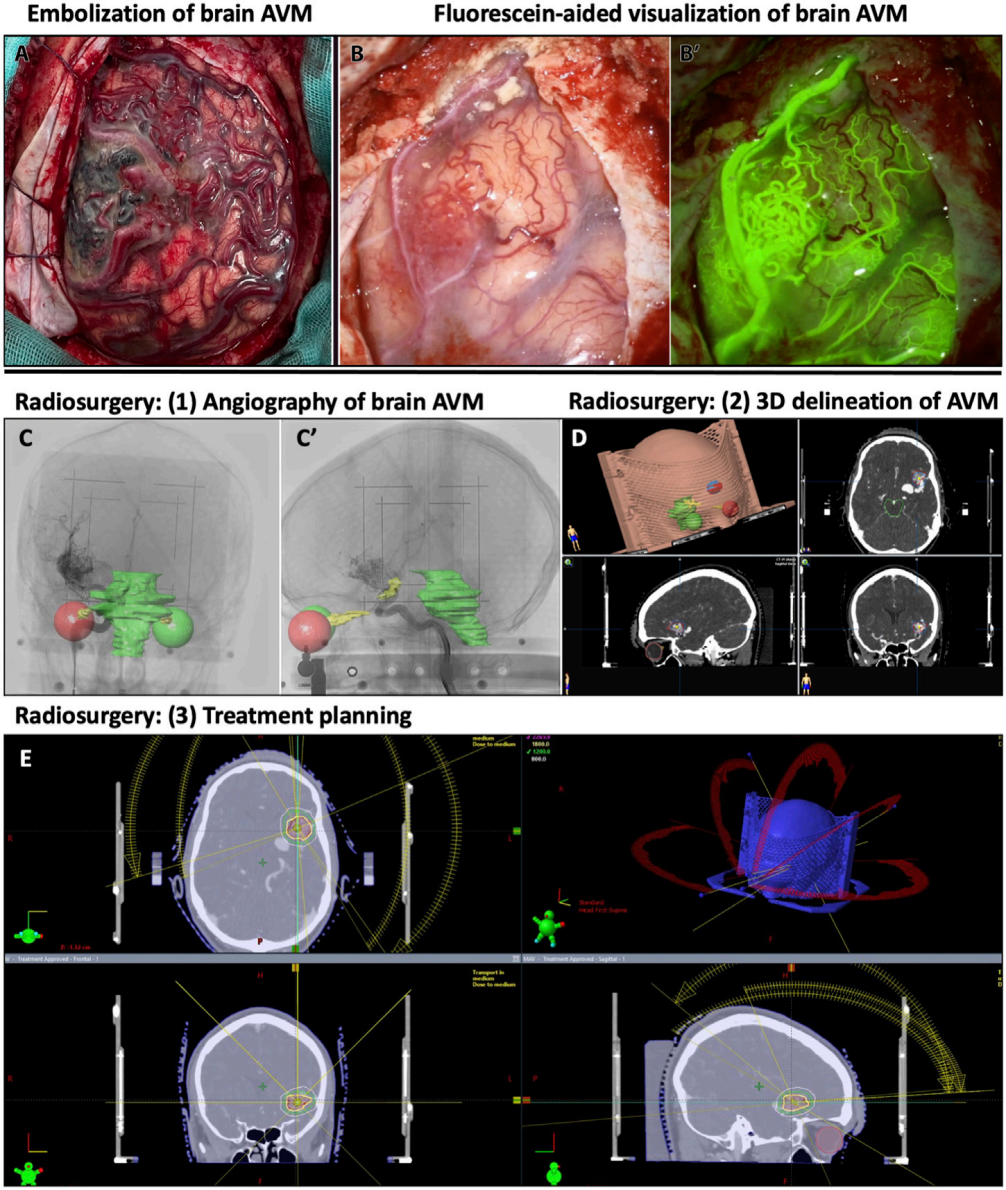
Examples of treatment and visualization methods for human brain AVMs. **(A)** Human brain with embolized AVM and surrounding vascular abnormalities. **(B-B′)** Human AVM on the frontal cortex, with obstructed view, and its corresponding fluorescein-perfused image for visualizing AVM nidus on human brain. **(C–E)** Radiosurgical planning to treat left sylvian AVM. **(C-C′)** Lateral and anteroposterior stereotactically-acquired angiography with radiosurgical sensitive structures delineated (visual pathways and brainstem). **(D)** 3D delineation of the AVM in radiosurgical planning. **(E)** Radiosurgical treatment planning with representation of the different radiation beams and different isodose lines around the treatment target.

Because of these alterations of the cerebral vasculature, brain AVMs can lead to seizures, headaches, stroke, hemorrhages, and neurological deficits (Nielsen et al., 2016; Solomon and Connolly, 2017; Scherschinski et al., 2022). Common methods to treat brain AVMs include embolization, radiation therapy, surgical resection of the unhealthy vasculature, or some combination thereof (Figure 2). However, these methods are not applicable to all brain AVM patients, as treatment options depend on AVM size, location, and on patient access to an AVM medical care team (Scherschinski et al., 2022). Given the heterogeneity of brain AVM presentation and angioarchitectural pathologies, there is controversy and longstanding debate over the best standard course of treatment for patients. For instance, combined treatment with embolization and radiosurgery can have differing results and success on patients with different AVM pathologies (Sackey et al., 2017). Repeated radiosurgery on AVMs can successfully resolve AVM pathologies but can leave the patient at higher risk for post-surgery complications (Kano et al., 2012). Amongst attempts to customize treatment plans for AVM patients, some clinical studies reported an increased rate of rupture in AVMs, thereby questioning the decision to pursue invasive treatments over medical management of symptoms (Luther et al., 2022). These findings have underscored the need for understanding patient and AVM heterogeneity and for developing and testing new treatments, such as targeted molecular or pharmacological therapies.

Research shows that brain AVMs affect vessel permeability and thus disrupt blood brain barrier (BBB) function (Hirunpattarasilp et al., 2019; Lendahl et al., 2019; Adhicary et al., 2023). Because brain capillaries (also known as microvessels) participate with other cell types as part of the neurovascular unit (NVU), it follows that brain AVMs lead to perturbed communication with other vascular cells (e.g., mural cells) and brain cells (e.g., astrocytes, neurons) (Winkler et al., 2022; Winkler et al., 2018; Chapman et al., 2022; Selhorst et al., 2022). Because NVU function is critical for brain homeostasis, including BBB integrity and neurovascular coupling, it is important to understand how NVU cells are affected during brain AVMs.

According to current statistics, less than 5% of brain AVMs have a known, heritable genetic lesion, while 95% of brain AVMs are currently considered to be sporadic—of those sporadic brain AVMs, a majority, but not all, appear to be associated with somatic mutations (Nikolaev et al., 2018; Adhicary et al., 2022). Genetic and RNA sequencing data from human brain AVMs have shown diversity in AVM phenotypes (Nikolaev et al., 2018; Huo et al., 2019; Hauer et al., 2020; Winkler et al., 2022). To understand the underlying diverse mechanisms of brain AVM formation and progression, many vertebrate models have been developed. Some models induce known genetic mutations identified in human patients, and other models target genes not yet known to cause brain AVM in humans. Pre-clinical mouse models are commonly used animal models that reliably reproduce features of brain AVM—AV shunting, nidus formation, hemorrhage, and abnormal blood flow—that are similar to those seen in humans (Figure 1C). Given these phenotypic similarities, research using mouse models has undoubtedly advanced our comprehensive understanding of brain AVMs and their genetic and cellular diversity.

### Spinal cord AVMs

Though CNS AVMs are more common in the brain, they can also occur in the spinal cord. In human patients, spinal cord AVMs account for approximately 25% of all vascular malformations identified in the spinal cord (Varshneya et al., 2019). Spinal cord vascular malformations are heterogenous, presenting with different anatomical pathologies and are classified into four subtypes, based on those vascular pathologies (Takai, 2017). Currently, spinal cord vascular malformations are classified as Type 1 (Spinal Dural Arteriovenous Fistula), Type 2 (Intramedullary Arteriovenous Malformation), Type 3 (Extradural-Intradural Arteriovenous Malformations), or Type 4 (Intradural Perimedullary Arteriovenous Fistula), depending on their location, symptoms, risk factors, and properties (Ferch et al., 2001; Patchana et al., 2020). Though classified as spinal vascular malformations, it is important to note that Type 1 and Type 4 are not true AVMs, but rather are vessel fistulas (Patchana et al., 2020). Type 2 AVMs are the most common type of spinal cord AVM, and they typically present as a nidus in the spinal cord (Ferch et al., 2001; Krings, 2010). Spinal cord AVMs may lead to hemorrhage and/or venous congestion (Krings, 2010) that can result in permanent disabilities (Hong et al., 2019). While spinal cord AVMs have been reported in mouse models (Milton et al., 2012), far less mechanistic insight has been gained, compared to brain AVMs. However the data from the few mechanistic studies that have been performed for spinal cord AVMs have shown that molecular expression of spinal cord AVMs is similar to retinal and brain AVMs (Vieira et al., 2020).

### Retinal AVMs

In animal models, AVMs are frequently observed in the retina. Retinal AVMs are generally reported as an enlarged AV connection together with other vascular defects. The retinas from animal models are widely used in AVM studies, due to their vascular anatomical simplicity (compared to brain tissue) and reproducibility. In mice, the retinal vasculature forms rapidly in the first weeks after birth (Mahmoud et al., 2010) resulting in a highly organized and hierarchical vascular network (Stahl et al., 2010; Crist et al., 2018). The simple and stereotyped artery-capillary bed-vein organization allows the presence of abnormal vasculature to be readily observed and imaged (Stahl et al., 2010). Further, because the retinal vasculature develops postnatally, this model can provide insight into AVMs that form during the early postnatal period (Stahl et al., 2010). Indeed, retinal vascular studies have been effectively translated to human and animal CNS AVMs.

### Hereditary Hemorrhagic Telangiectasia associated AVMs

HHT is an inherited disorder in which vascular abnormalities, including AVMs, occur in multiple parts of the body (Kim et al., 2018; Snodgrass et al., 2021). In HHT patients, AVMs can occur in multiple organs, including lung and brain (Thalgott et al., 2015; Kim et al., 2018; Snodgrass et al., 2021). Genetic studies revealed that most commonly, germline mutations in *endoglin* (*ENG*) or *activin A receptor like Type 1* (*ACVRL1*) cause HHT1 or HHT2, respectively (Johnson et al., 1996; Mahmoud et al., 2010; Choi et al., 2014; Tual-Chalot et al., 2014; Thalgott et al., 2015; Baeyens et al., 2016; Ola et al., 2018). HHT animal models, which disrupt Endoglin and Alk1 (encoded by *ACVRL1*) signaling pathways, have been developed to study brain AVM onset and progression. These models have proven very useful for uncovering mechanisms of brain AVM pathogenesis and relating those findings to other brain AVM models.

## Mural cells of the CNS

Mural cells are specialized cell types associated with endothelial cells in a vascular network. Mural cells are juxtaposed to endothelial cells and play important roles in vascular development and maintenance, vessel stability, and blood flow regulation (Uemura et al., 2020). The term “mural cells” is an umbrella term that encompasses both pericytes and vascular smooth muscle cells (SMCs). The roles of mural cells during CNS AVM are not well understood; however, numerous studies have shown that CNS AVMs are associated with molecular, cellular, and functional changes in mural cells (Tables 1, 2).

**TABLE 1:** Changes to pericytes in mouse CNS AVM models.

Genetic model	CNS location	Location of genetic manipulation	Genetic manipulation timepoint	Cellular characteristics of pericytes	Molecular characteristics of pericytes	Observed phenotype timepoint	AVM?	References
*Cdh5-CreER^T2^; Rbpj^flox/flox^*	Brain	ECs	P1, P2 (tamoxifen injection)	Increased PC area. Unchanged PC coverage	Decreased expression of Pdgfrβ, *N-Cadherin, CD146*	P10, P14, P21	Yes	Selhorst et al. (2022)
				Increased cellular projections (P14, P21). Unchanged proliferation. Increased number of PCs				
						P14		
	Retina			Increased PC area. Unchanged PC coverage		P10, P14	Abnormal vasculature	
*Cdh5-CreER^T2^; Alk1^flox/flox^*	Retina	ECs	P3 (tamoxifen injection)	Altered PC recruitment. Decreased PC coverage in vascular plexus. Unaffected PC coverage in sprouting front	///	P5	Yes	Baeyens et al. (2016)
	Retina	ECs	P4 (tamoxifen injection)	Decreased PC coverage	///	P6	Yes	Tual-Chalot et al. (2014)
*Alk1^1flox/2flox^*	Brain	ECs	8 weeks (Ad-Cre and AAV-VEGF injection)	Decreased PC coverage	Decreased expression of *Pdgfrβ*	16 weeks	Yes	Chen et al. (2013)
*Alk1^2flox/2flox^*				Reduced PC number				Zhu et al. (2018)
*Cdh5-CreER^T2^; Smad4^flox/flox^*	Retina	ECs	P1, P4 (tamoxifen injection)	Decreased PC coverage	Decreased expression of *NG2 and Desmin*	P7	Yes	Crist et al. (2018)
	Retina	ECs	P0, P1, P2 (tamoxifen injection)	Decreased PC coverage	///	P6	Yes	Ola et al. (2018)
*Pdgfrβ-CreER^T2^; Rbpj^flox/flox^*	Retina	PCs	P1, P2, P3 (tamoxifen injection to nursing mom or pups)	Decreased PC coverage	///	P10, P14, 6 weeks	Yes (starting at P14)	Nadeem et al. (2020)
				Decreased PC coverage of endothelium at angiogenic front	Decreased expression of *Pdgfrβ* (at P6)	P5	No	
	Brain		E9.5, E10.5, E11.5 (tamoxifen injection to dams)	Reduced PC coverage of endothelium	///	E18.5	Abnormal Vasculature	
	Brain	PCs	P1, P2, P3 (tamoxifen injection)	Abnormal PC morphology. Increased cellular projections and membrane perfusions	Decreased expression of *PdgfrB, Anpep, Rgs5, Cspg4, Notch3* and 30 other downregulated PC markers via scRNA-seq	P10	Abnormal Vasculature (Increased vessel diameter)	Diéguez-Huertado et al. (2019)
				Increased contractility of PCs	*Increased expression of Acta2 (corr. in vitro), Tagln (corr. in vitro), Serpine 1, Ctgf, Tgfβi,Timp1, Postn, Tnc, MMPs, TgfB3, Thbs1,αSMA, SM22α, Vimentin, Desmin, Nestin, pSMAD3*			
				Unchanged PC coverage	Altered expression of integrin α subunits			
*Pdgfrβ-CreER^T2^; Notch1^+/+^; Notch3^flox/flox^*	Retina	PCs	P1, P2, P3 (tamoxifen injection)	Reduced PC coverage	///	P14	Abnormal Vasculature (Increased vessel diameter)	Nadeem et al. (2020)
*Pdgfrβ-CreER^T2^; Notch1^flox/+^; Notch3^flox/flox^*; *Pdgfrβ-CreER^T2^; Notch1^flox/flox^; Notch3^flox/flox^*							Yes	
*Pdgfrβ-CreER^T2^; dnMAML^flox/flox^*	Retina	PCs	P1,P2, P3 (tamoxifen injection)	Decreased PC coverage	///	6 weeks	Yes	Nadeem et al. (2020)
*Pdgfrβ-CreER^T2;^ Srf^flox/flox^*	Retina	PCs and vSMCs	P1, P2, P3 (tamoxifen injection)	Decreased PC coverage	Decreased expresion of *F-actin, Actb*	P6	Yes (at P12)	Orlich et al. (2022)
				Decreased PC migration (*in vitro*)				
				Abnormal PC morphology. Abnormal PC processes (short and stubby)				
				Partial detachemnt from endothelium				
				No longer able to form filopodia				
*Notch1^+/−^; Notch3^−/−^*	Retina	Germline Deletion		Unchanged PC number. Decreased PC coverage. Abnormal PC morphology. Abnormal PC processes. PC dissociation from endothelium. Altered PC-EC interaction	Altered MMPs (*in vitro*). Decreased expression of *Pdgfrβ* (corr. *in vitro*)	P5	Abnormal Vasculature (AVMs at P13)	Kofler et al. (2015)
*Notch3^−/−^*	Retina	Germline Deletion		Decreased PC coverage. Abnormal PC morphology. Abnormal PC processes. Decreased PC number	Decreased expression of *Pdgfrβ*	P5	Abnormal Vasculature	
*Notch1^+/−^*	Retina	Germline Deletion		Decreased PC coverage. Unchanged PC number	Decreased expression of *Pdgfrβ*	P5		

EC, endothelial cell; PC, pericyte, Corr; corroborated; pSMAD3, phosphorylated SMAD3; Alk1, Acvrl1; Adenovirus-Cre, Ad-Cre; adeno-associated virus-VEGF, AAV-VEGF; ///, not reported or does not apply.

**TABLE 2:** Changes to smooth muscle cells in mouse CNS AVM models.

Genetic model	CNS location	Location of genetic manipulation	Genetic manipulation timepoint	Cellular characteristics of SMCs	Molecular characteristics of SMCs	Observed phenotype timepoint	AVM	References
*Alk1^1flox/2flox^*	Brain	ECs	8 weeks (Ad-Cre and AAV-VEGF injection)	///	Decreased expression of αSMA	16 weeks	Yes	Chen et al., 2013
*Alk1^2flox/2flox^*								Zhu et al. (2018)
*Cdh5-CreERT2; Smad4^flox/flox^*	Retina	ECs	P0, P1, P2 (tamoxifen injection)	Decreased arterial αSMA. Increased venous αSMA coverage	///	P6	Yes	Ola et al. (2018)
	Retina	ECs	P1, P4 (tamoxifen injection)	Impaired vSMC coverage in veins and AVMS	Increased αSMA expression in veins and AVMs	P7	Yes	Crist et al. (2018)
*Cdh5-CreERT2; Eng^2flox/2flox^*	Retina	ECs	P2, P4 (tamoxifen injection for neonates)	Abnormal vSMC organization	Decreased expression of αSMA.	P6	Yes (by P7)	Mahmoud et al. (2010)
*Pdgfrβ-CreERT2; Rbpj^flox/flox^*	Retina	PCs	P1, P2, P3 (injection to nursing mom or pups)	Decreased vSMC coverage of arteries	Increased αSMA expression in veins	P10, P14, 6 weeks	Yes	Nadeem et al. (2020)
				Reduced vSMC coverage of arteries	No αSMA expression in veins	P5	No	
					Decreased αSMA expression in arteries			
	Brain	PCs	P1, P2, P3 (tamoxifen injection)	Reduced vSMC coverge	Decreased expression of Acta2, Tagln	P10	Yes	Diéguez-Huertado et al. (2019)
*Pdgfrβ-CreER^T2^; Notch^1+/+^; Notch3^flox/flox^*	Retina	PCs	P1, P2, P3 (tamoxifen injection)	Reduced vSMC coverage	///	P14	Abnormal Vasculature (Increased vessel diameter)	Nadeem et al. (2020)
*Pdgfrβ-CreER^T2^; Notch1^flox/+^; Notch3^flox/flox^*							Yes	
*Pdgfrβ-CreER^T2^; Notch1^flox/flox^; Notch3^flox/flox^*					Increased αSMA expression in veins			
*Pdgfrβ-CreER^T2^; dnMAML^flox/flox^*	Retina	PCs	P1, P2, P3 (tamoxifen injection)	Decreased vSMC coverage	///	6 weeks	Yes	Nadeem et al. (2020)
*Pdgfrβ-CreER^T2^; Srf^flox/flox^*	Retina	PCs and vSMCs	P1, P2, P3 (tamoxifen injection)	Hierarchical patterning defects. Dilated artery and veins. Reduced vascular bed, decreased vessel branch points	Decreased expression of αSMA*(Acta2), Mhy11, Tagln, Myl9, Tpm2, Actc1, Actg1, Cacna1d, Cacna1f, Kcnma1, Kcnmb1*	P12	Yes	Orlich et al. (2022)
				Increased venous vSMC and decrased arterial vSMC coverage				
				Reduced vSMC contractility				
*SM22α-Cre; Alk1^flox/flox^*	Brain, Spinal Cord	SMCs	with SM22α-Cre expression	Abnormal vSMC coverage	///	18–102 weeks	Yes	Milton et al. (2012)
*SM22α-Cre; Alk1^flox/-^*								
*SM22α-Cre; Eng^2flox/2flox^*	Brain, Spinal Cord	SMCs	with SM22α-Cre expression	///	///	5 weeks	Yes	Choi et al. (2014)
*Notch1^+/−^; Notch3^−/−^*	Retina	Germline Deletion		Impaired vSMC differentiation	Decreased expression of αSMA	P5	Abnormal Vasculature (AVMs at P13). Abnormal Vasculature	Kofler et al. (2015)
*Notch3^−/−^*								

EC, endothelial cell; PC, pericyte; Corr, corroborated; pSMAD3, phosphorylated SMAD;, Alk1, Acvrl1; Adenovirus-Cre, Ad-Cre; adeno-associated virus-VEGF, AAV-VEGF; ///, not reported or does not apply.

### Pericytes

Pericytes are mural cells that are found in most microvascular beds in the vascular system; however, the density and coverage of pericytes on microvessels varies among different tissue types (Armulik et al., 2011). In the CNS, the pericyte:endothelial cell ratio is estimated to be between 1:1 and 1:4, making the CNS microvasculature one of the most pericyte-enriched systems (Armulik et al., 2011; Smyth et al., 2018). Pericytes generally have a small cell body with multiple long, thin cytoplasmic processes that enwrap the underlying microvessel (Hill et al., 2015). One pericyte can extend its processes to many endothelial cells, permitting intercellular communication and influences with those endothelial cells (Gerhardt and Betsholtz, 2003).

Pericytes are critical cells within the CNS NVU. Here, at the level of the microvasculature, cells of the NVU collectively contribute to BBB formation and function. The BBB is inherently restrictive and selective, when determining what solutes are exchanged between the microvasculature and the CNS parenchyma. Of blood barriers in mammalian physiology (e.g., blood-cerebrospinal fluid, blood-testis, blood-retina barriers), the BBB is the most restrictive. Cell-cell junctions between neighboring endothelial cells provide barrier selectivity. However, endothelial cells are not the only NVU cells that contribute to BBB stringency. Specialized features of other NVU cells, like pericytes, play critical roles in development, regulation, and maintenance of tight junctions (Luissint et al., 2012), and thus promote BBB integrity. Pericytes and endothelial cells have a close cellular relationship, sharing a basement membrane and communicating with one another through multiple different signaling pathways (Adhicary et al., 2022). Pericytes communicate with endothelial cells through paracrine signaling, by diffusion of small molecules or, in some locations, via direct contact to allow for intercellular communication along a capillary segment (Adhicary et al., 2022). In fact, pericyte and endothelial cell communication is critical for proper formation and maintenance of the BBB. For example, pericytes can influence gene expression in endothelial cells that regulate vessel permeability (Freitas-Andrade et al., 2020).

Though the roles of pericytes can be generalized, it is important to note that pericytes are heterogenous in nature. CNS pericytes not only differ in their molecular and cellular profiles, when compared to other vascular beds (Su et al., 2021), but CNS pericytes also differ from one another and may be parsed into pericyte subtypes, based on morphological, molecular, and functional profiles (Brown et al., 2019). Within the CNS, different vessel segments have different types of pericytes with varying functions (Grant et al., 2019; Uemura et al., 2020). For example, pericytes that exist near high-flow vessel segments (such as arterioles) are broad cells that use their surface area to enwrap the vessel; which can have α-smooth muscle actin (α-SMA) fibers with greater contractile properties, perhaps to regulate blood flow and blood routing (Brown et al., 2019; Erdener et al., 2022; Hartmann et al., 2022). By contrast, thin-strand, mid-capillary pericytes extend longer, strand-like processes along the length of a vessel, spanning and contacting several endothelial cells. These pericytes can exhibit thin α-SMA fibers, perhaps to permit communication between cells and to promote blood brain barrier integrity, at the microvascular level (Erdener et al., 2022; Hartmann et al., 2022).

Contractile function of pericytes is a recent field of study, and we are beginning to learn how the role of contractile pericytes can influence vascular disease outcomes; for example, contraction of brain pericytes after stroke has been associated with capillary flow stalling and capillary dysfunction (Staehr et al., 2023).

Pericytes have also been reported to have stem-like cell properties that allow them to retain their potency and differentiate into other cell types, depending on the physiological situation (Özen et al., 2014; Nakagomi et al., 2015; Brown et al., 2019; Farahani et al., 2019). Though the mechanisms that trigger pericyte transdifferentiation are not clear, a few CNS AVM models suggest that pathological changes in pericytes—molecular and morphological changes—trigger pericytes to acquire SMC characteristics, such as contractile properties (Diéguez-Hurtado et al., 2019; Selhorst et al., 2022) (Table 1).

Pericytes can be affected either directly or indirectly during CNS AVMs. Genetic mutations in pericytes can lead to formation of AVMs, or mutations in other vascular cells (such as endothelial cells) can have indirect effects on pericytes, which may exacerbate AVM pathology (Table 1). One focus of this review is to describe direct and indirect changes to pericytes—including molecular, cellular, and functional changes—in the context of CNS AVMs. These changes are discussed in the text and are compiled in the Tables. In Table 1, we compiled a list of studies reporting changes to pericytes in mouse CNS AVM tissue. As our understanding of pericyte heterogeneity advances, so too will our understanding of how these cells are affected during CNS AVMs.

### Vascular smooth muscle cells

**FIGURE 3:**
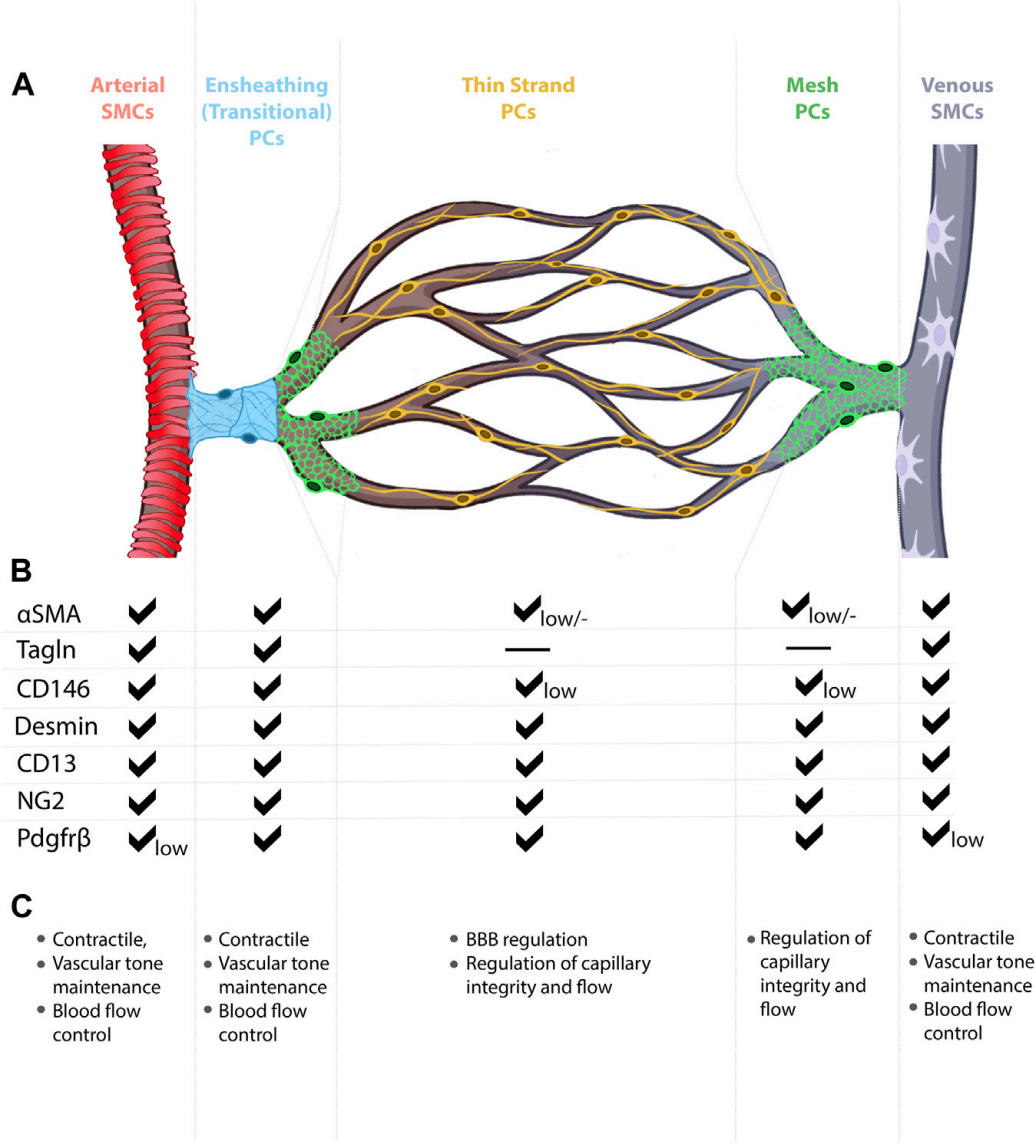
Mural cell heterogeneity in the mammalian brain vasculature. **(A)** Capillary network from artery to vein with representative types of mural cells for each vessel segment. **(B)** Mural cell subtypes express different combinations of molecular markers, at different expression levels. **(C)** Known and predicted functions for mural cell subtypes.

Within the CNS, perivascular SMCs enclose the endothelial tube of arteries, arterioles, some post-capillary venules, and veins, but are absent in capillaries (Uemura et al., 2020). Arteriolar SMCs are contractile and can regulate blood flow and pressure through vasoconstriction and vasodilation (Hill et al., 2015; Aldea et al., 2019). Contractility of arterial SMCs also maintains healthy vascular tone, in response to blood pressure changes and shear stress, and maintains arterial diameter within a normal physiological range (Aldea et al., 2019). In general, arteriolar SMCs are situated perpendicular to blood flow and vessel length and parallel to adjacent SMCs. SMC morphology differs, depending on the type of vessel on which they lie. Compared to arteriolar SMCs, venular SMCs have a nonuniform, stellate shape (Figure 3). Venous SMCs play important roles in maintaining venous blood volume depending on cardiac output by vasodilation and vasoconstriction (Downey and Heusch, 2001; Sykora et al., 2016). In Table 2, we have assembled a list of studies reporting changes to SMCs in mouse CNS AVM tissue.

### Perivascular fibroblasts

Perivascular fibroblasts have recently come into focus in the brain vasculature as a cell type located in the arteries, between the vessel wall and astrocytic endfeet and more loosely attached to the vessels, as compared to pericytes or SMCs (Vanlandewijck et al., 2018). Perivascular fibroblast morphology is distinct from other perivascular cells, as they have a flat soma and non-overlapping processes (Vanlandewijck et al., 2018; Dorrier et al., 2022). Perivascular fibroblasts and fibromyocytes have recently been implicated in human CNS AVM samples (Winkler et al., 2022). While it was suggested that SMCs may differentiate into perivascular fibromyocytes, evidence for such cellular transdifferentiation has not yet been reported (Winkler et al., 2022).

## Mural cells in human AVM

The study of mural cells in human AVMs is limited but is gaining momentum in the field. Ongoing studies focus on expression changes in resected AVM tissue and cells. In this regard, expression profile data have been acquired using immuno-based methods and RNA sequencing methods. These methods have uncovered cell-specific gene and protein expression changes in AVM *versus* healthy tissue. Mural cells have become more prominent in these reports in the last decade. In Table 3, we compiled a list of studies reporting changes to mural cells in human CNS AVM tissue. Clinical observations to date have shown abnormal mural cell proliferation, coverage of microvessels, and cellular differentiation (Table 3). To gain deeper mechanistic understanding of how mural cells are affected in AVMs, and how mural cells may be driving AVM pathologies, animal models have been developed. With increased focus on mural cells in AVMs, it is becoming increasingly clear that there are no simple phenotypic descriptions of consequences to mural cells that can be applied to *all* AVM pathologies. Here, we highlight both mural cell heterogeneity and AVM heterogeneity, with the hope that CNS AVM pathologies can begin to be understood, at least in part, in terms of the types of mural cells affected and how those mural cells are affected. Tables 1–3 outline current data regarding mural cells in animal models of CNS AVMs and in experiments with resected human AVM tissue. As animal models have proven effective at recapitulating important features of CNS AVMs, the reported effects to mural cells may indeed prove relevant to mechanisms of human AVMs and to potential therapeutic avenues.

**TABLE 3:** Changes to mural cells in human CNS AVMs.

Method	Source	CNS location	# of samples	Cellular characteristics of perivascular cells in AVM	Molecular characteristics of perivascular cells in AVM	References
Surgical resection of AVM	Human Patients	Brain (Temporal Lobe)	20 AVM Samples	Reduced pericyte number. Decreased pericyte coverage	///	Winkler et al. (2018)
		Brain	8 AVM Samples	///	Increased Notch4 expression in SMCs	ZhuGe et al. (2013)
		Brain	43 AVM Samples	Increased SMC Proliferation	Increased αSMA expression. SNAI1/2/protein expression in SMCs. Altered cytokine expression. Increased PAI-1 protein expression	Shoemaker et al. (2020)
		Extracranial and Brain	9 AVM Samples	Abnormal αSMA coverage on arteries	///	Davis et al. (2018)
		Brain	5 AVM Samples	Altered vSMC differentiation	Reduced expression of *smoothelin*	Uranishi et al. (2001)
		Brain	15 AVM Samples	Decreased number of pericytes	///	Tu et al. (2006)
		Brain, human vSMCs	5 AVM Samples (scRNAseq)	///	Increased HIGD1B expresison in PCs. Increased expression of *metallothionein*s in SMCs	Winkler et al. (2022)
				Perivascular fibroblasts and fibromyocytes present		
		Brain	51 AVM Samples	///	Increased expresison of *FZD10* and *MYOC* in SMCs from LFR bAVMs	Huo et al. (2019)
		Brain	8 AVM Samples	///	Increased *Notch3* expression in bAVM SMCs	Hill-Felberg et al. (2015)
Cell Culture	k-RasV12 ECs, Human brain vascular pericytes	///	///	Abnormal pericyte recruitment. Decreased number of pericytes. Decrease in periocyte elongation. Perturbed pericyte-EC communication	///	Sun et al. (2022)

///, not reported or does not apply; LFR, low flow rate.

## Signaling pathways in CNS AVMs

**FIGURE 4:**
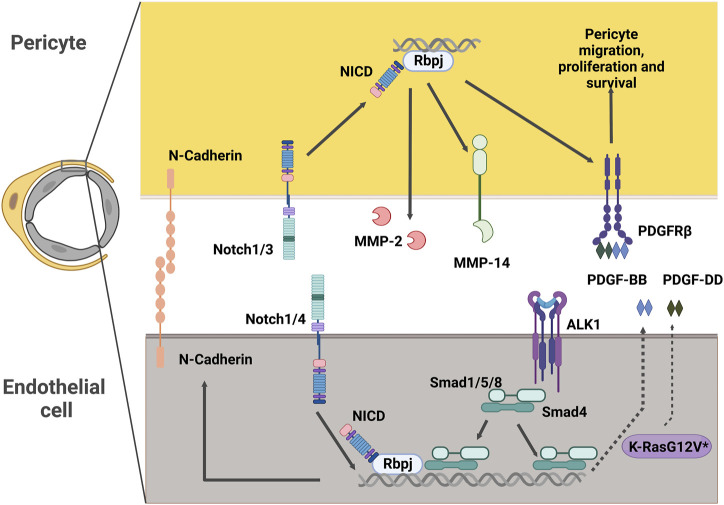
Potential intercellular signaling between endothelial cells and pericytes in CNS AVMs. Notch signaling in pericytes promotes expression of MMP-14 and MMP-2, which are modulators of extracellular matrix structure. Notch signaling in pericytes also promotes expression of PDGFRβ, a key receptor driving essential pericyte functions such as migration, proliferation and survival. In endothelial cells, Notch intracellular domain can associate with Smad4 to regulate the levels of endothelial N-Cadherin. Alk1 signals in endothelial cells to promote the expression of PDGF-B. Expression of the pathogenic variant K-RasG12V in endothelial cells results in higher levels of PDGF-D. Both PDGF-B and PDGF-D function as ligands of the PDGFRβ present in mural cells. NICD: Notch Intracellular Domain, MMP: Matrix Metalloprotease, PDGFR: Platelet Derived Growth Factor Receptor, PDGF: Platelet Derived Growth Factor, ALK1: Activin receptor-like kinase 1, Rbpj: Recombination signal binding protein for immunoglobulin kappa J.

Within the CNS vasculature, endothelial cells and mural cells are juxtaposed and thus anatomically positioned to communicate with and influence one another. Molecular signaling pathways between endothelial cells and pericytes, for example, foster intercellular interactions to maintain vascular development and homeostasis (Brown et al., 2019). Research has shown that multiple different signaling pathways are affected in CNS AVMs, thereby offering insight into molecular mechanisms of the disorder. Here, we describe some of the most relevant signaling pathways involved in mural cell-endothelial cell communication in the context of CNS AVMs (Figure 4).

### PDGFRβ signaling pathway

The Platelet Derived Growth Factor (PDGF) family includes a series of multifunctional factors acting fundamentally in stromal cells. PDGF ligands signal through the cell-surface tyrosine kinase receptor PDGFR. The most relevant *in vivo* interaction of PDGFs with their receptors in the vascular system is that of PDGF-BB and PDGFRβ (Andrae et al., 2008). PDGF-BB is secreted by endothelial cells and signals through PDGFRβ in pericytes to regulate key functions in these cells, including recruitment, proliferation, and survival (Armulik et al., 2011). Consequently, mice lacking PDGF-BB or PDGFRβ exhibit a dramatic absence of pericytes. This paucity of pericytes results in increased capillary diameter, endothelial hyperplasia (Hellström et al., 2001), and microaneurysms (Lindahl et al., 1997), thus highlighting the importance of pericyte-endothelial cell interactions in the regulation of vascular diameter.

Interestingly, absence of pericytes and pericyte coverage has been reported in human samples of brain AVMs (Winkler et al., 2018) and in models of the disease (Chen et al., 2013; Tual-Chalot et al., 2014; Kofler et al., 2015; Baeyens et al., 2016; Crist et al., 2018; Ola et al., 2018; Nadeem et al., 2020; Orlich et al., 2022). With this evidence, it is tempting to speculate that loss of function mutations in the PDGF-BB/PDGFRβ axis might be associated with the development of AVMs. As a matter of fact, pathogenic variants of *PDGFRB* have been described associated with fusiform cerebral aneurysms (Karasozen et al., 2019; Chenbhanich et al., 2021; Parada et al., 2022) and in a small subset of patients with brain AVMs (Gao et al., 2022). Surprisingly, the pathogenic variants identified as associated with brain aneurysms result in gain of function of PDGFRβ (Wenger et al., 2020). Whether the already identified pathogenic variants in brain AVMs also lead to gain of function of the PDGFRβ, and how prevalent they are, remains to be established.

### Notch signaling pathway

Notch signaling is a ubiquitous signaling pathway regulating critical functions in health and disease. Notch receptors (1–4) in the surface of the cell interact with the ligand (Delta-like 1, 3, 4 or Jagged 1, 3) in the neighboring cell. This interaction of Notch with its ligand creates a conformational change in the Notch receptor, exposing it for cleavage by ADAM metalloproteases and the γ-secretase complex and causing the release of the Notch intracellular domain (NICD). The NICD translocates to the nucleus where it binds to Recombination signal binding protein for immunoglobulin kappa J region (Rbpj) and a series of other co-activator factors to promote the transcription of its target genes (Siebel and Lendahl, 2017).

In the vasculature, endothelial cells express mostly Notch 1 and 4, while mural cells express Notch 1, 2, and 3. Of the ligands, Delta-like1 (Dll1), Dll4, and Jagged1 (Jag1) are the most prevalent in the endothelium, with Jag1, Dll4, and to some degree Dll1 and Jag2, also being found on mural cells (Hofmann and Iruela-Arispe, 2007; Baeten and Lilly, 2015; He et al., 2018). Consequently, Notch signaling in blood vessels has a wide range of functions mediated by ligand-receptor crosstalk between endothelial and mural cells (Gridley, 2010; Del Gaudio et al., 2022; Hasan and Fischer, 2023).

Notch signaling in the vasculature has been widely associated with brain AVMs in human patients and mouse models of the disease (Drapé et al., 2022). The first insights for a role of Notch signaling in AVM formation come from studies in embryos with mutations in different molecules of the Notch signaling pathway. Injection of India ink in mutants haploinsufficient for the Notch ligand *Delta-like 4* (*Dll4*) or with deficiency of endothelial *Rbpj* revealed enlarged AV shunts. A few years later, it was reported in postnatal mice that Notch gain of function in endothelial cells gave rise to AVM formation, and together with further evidence from mutant embryos (Krebs et al., 2010), it became apparent that both gain and loss of function of endothelial Notch signaling can lead to AVM formation in mouse models. However, these early studies did not document whether AVMs were arising in the brain. Additional work using mouse models to activate (Murphy et al., 2008; Murphy et al., 2012; Murphy et al., 2014; Nielsen et al., 2023) or suppress Notch signaling (Nielsen et al., 2014; Selhorst et al., 2022; Adhicary et al., 2023) in endothelial cells was essential to pinpoint the role of Notch in brain AVMs.

Because of the strong requirement for Notch signaling in endothelial cells to prevent the development of brain AVMs, changes in the mural cell compartment were frequently overseen or not documented in the different mutants described above. In another study of *Rbpj* deletion from endothelial cells, numerous pericyte abnormalities were seen in brain and retina AVMs, including abnormal morphology and increased pericyte area with unchanged pericyte coverage of vessels (Selhorst et al., 2022). In this AVM model, pericytes expressed reduced levels of *Pdgfrb*, *Cdh2*, and *Cd146* (Selhorst et al., 2022), suggesting that, in addition to preventing AVMs, endothelial Rbpj controls the expression of factors promoting pericyte recruitment and association with endothelial cells. Similar, yet somewhat contradictory, results were obtained by Li et al., (2011), who reported that loss of endothelial *Rbpj* using a different Cre-driver also resulted in reduced *Cdh2* expression. However, while this study documented loss of pericytes, it did not document the formation of AVMs.

The function of Notch in CNS AVM formation was also evaluated using mice with germline deletion of one allele of *Notch1,* together with deletion of one or both alleles of *Notch3* (Kofler et al., 2015). Abnormal, hyperdense vasculature was observed in *Notch1* ^*+/−*^and *Notch3^−/−^* mice; however, AVMs were only observed in the double mutant mice *Notch1* ^*+/−*^ *; Notch3^−/−^* mice. It was not clear from these studies whether Notch1 and Notch3 function in the mural cell compartment, in the endothelium, or in some combination thereof, was responsible for preventing AVM development. Subsequent studies, which impaired Notch signaling specifically in mural cells, demonstrated a role for Notch signaling in pericytes to prevent the development of retinal AVMs (Nadeem et al., 2020). Mechanistically, the Notch pathway prevents AVM onset by regulating the levels of Matrix Metalloprotease 14 (Kofler et al., 2015) and PDGFRβ (Kofler et al., 2015; Nadeem et al., 2020) in pericytes. Intriguingly, the work of Diéguez-Hurtado et al., (2019), using mouse models with mural cell deficiency of *Rbpj*, describes a Notch independent role for Rbpj in pericytes where vascular AV shunts are detected. However, these lesions lack the characteristic fast-flow associated with AVMs, and the overall lesions in this mouse model are described to resemble cerebral cavernous malformations (CCMs).

In addition to findings from animal models, dysregulated NOTCH signaling in mural cells has also been observed in brain AVM samples from human patients (ZhuGe et al., 2009; ZhuGe et al., 2013; Hill-Felberg et al., 2015). Upregulation of NOTCH4 and NOTCH1 was detected in both endothelial cells and SMCs in human brain AVM samples (ZhuGe et al., 2013; ZhuGe et al., 2009). Similarly enhanced levels of NOTCH4 were also reported by Hill-Felberg et al., (2015); however, these authors highlighted that upregulation of NOTCH3, expressed by endothelial cells and mural cells, was substantially more robust than that of NOTCH4 and that levels of NOTCH1 were decreased in brain AVMs samples.

It is intriguing that loss of Notch signaling in mural cells results in AVM formation in animal models, yet Notch signaling is mostly found to be upregulated in the mural cell compartment of human brain AVM samples. Further studies are needed to carefully and thoroughly delineate how Notch in pericytes and SMCs impacts CNS AVM formation and/or progression.

### BMP/ALK/SMAD pathway

Bone Morphogenetic Proteins (BMPs) are soluble factors which belong to the Transforming Growth Factor-β (TGFβ) superfamily (Wrana, 2013). Several BMP members play important roles in the vasculature (Kulikauskas et al., 2022). BMP9 and BMP10, in particular, form a distinct subgroup within the TGFβ superfamily. These factors are released into the circulation from the liver and the heart, respectively, and signal through the activin receptor-like kinase 1 (Alk1)/BMPRII complex (David et al., 2007). This signal can be regulated by the co-receptor Endoglin, which interacts with the complex to promote phosphorylation of Smad1/5/8, resulting in their release, association with Smad4, and further translocation to the nucleus to regulate the expression of their target genes (Ross and Hill, 2008).

Mutations in the components of this pathway are associated with HHT and the development of AVMs. As such, mutations in the genes encoding for ENDOGLIN (*ENG*) and ALK1 (*ACVRL1*) result in the pathogenesis of HHT in 90% of the patients (McAllister et al., 1994; Johnson et al., 1996). Less common mutations in *SMAD4* lead to a particular type of HHT, known as juvenile polyposis (JT)-HHT (Gallione et al., 2004).

The familial nature of HHT and identification of causal genes has facilitated the generation of several mouse models recapitulating the disease (Arthur and Roman, 2022). Interestingly, endothelial deletion of *Alk1* or *Smad4* leads to the formation of AVMs in the retina (Tual-Chalot et al., 2014; Baeyens et al., 2016; Crist et al., 2018; Ola et al., 2018) or the brain (Chen et al., 2013), which display decreased pericyte coverage, indicating a likely contribution of this cell type toward preventing the onset or progression of the lesions. Supporting this idea, treatment of *Eng*-deficient mice with Thalidomide ameliorated retinal AVM severity, in part, by increasing mural cell coverage (Lebrin et al., 2010).

Several studies have reported crosstalk between the Notch signaling pathway and the BMP/ALK/Smad pathway. During angiogenesis, ALK1 and Notch signaling pathways synergize to promote the expression of Notch target genes and repression of endothelial sprouting (Larrivée et al., 2012; Moya et al., 2012; Ricard et al., 2012; Kerr et al., 2015; Rostama et al., 2015). In mouse models of AVMs, in which BMP/ALK1/Smad signaling is impaired, Notch signaling was also downregulated (Yao et al., 2011; 2013; Tual-Chalot et al., 2014; Hwan Kim et al., 2020). Interestingly, *Alk1* deficient HHT zebrafish models were not influenced by changes in the Notch signaling pathway, indicating that Notch signaling may not play a critical role in HHT associated AVMs (Rochon et al., 2015).

Most of the evidence gathered from mouse models of HHT has led to the general consensus that vessel abnormalities arise from loss of gene function in endothelial cells (Arthur and Roman, 2022). However, a role for the BMP/ALK1 axis in mural cell function has been established (Wang et al., 2021). More importantly, deletion of *Eng/Alk1* in mural cells, using the SM22α-Cre driver to target mural cells, leads to the development of AVMs in the brain of mice (Milton et al., 2012; Choi et al., 2014; Han et al., 2022). However, studies have suggested that the SM22α-Cre driver targets a small population of endothelial cells, which might be responsible for the observed phenotypes. Moreover, alternative mouse models where *Eng/Alk1* deletion was induced using other mural cell drivers (Ng2-CreER or Myh11-CreER^T2^) did not result in AVMs (Chen et al., 2014; Garrido-Martin et al., 2014). Additional evidence is needed to establish a clear role for the BMP/ALK/SMAD axis in mural cells during the onset or progression of CNS AVMs.

### RAS/MAPK pathway

The majority of CNS AVMs are sporadic and not linked to an inherited gene mutation. Recently, activating mutations in *KRAS* were uncovered in a high percentage of sporadic brain AVMs (Nikolaev et al., 2018). Thus far, the pathogenic variants of KRAS leading to brain AVMs seem to be restricted to endothelial cells and are not detected in the mural cell compartment (Nikolaev et al., 2018). The absence of pathogenic variants in mural cells is consistent with the data obtained using mouse models, in which expression of the pathogenic variant *KrasG12D* in endothelial cells is sufficient to recapitulate the phenotype observed in human lesions (Fish et al., 2020).

Further insight on the consequences of KRAS pathogenic variants in endothelial cells, and their interaction with pericytes, comes for co-culture experiments where Human Umbilical Endothelial Cells (HUVECs) expressing the pathogenic variant *KRASG12V* were cultured together with Human Brain Vascular Pericytes (HBVPs). *KRASG12V* expressing HUVECs formed abnormal vascular tubes with reduced pericyte coverage and reduced pericyte derived basement membrane, compared to control HUVECs (Sun et al., 2022). Among the most common factors that are produced by endothelial cells, yet control pericyte functions, PDGF-D and TGFβ2 expression was reduced in *KRASG12V* expressing HUVECs. PDGF-DD, similar to PDGF-BB, can drive pericyte migration and proliferation (Kemp et al., 2020). Future experiments will determine whether levels of pericyte coverage/numbers are also diminished in brain AVMs arising from the *KRASG12V* pathogenic variant in samples from human patients, whether levels of PDGF-DD are altered, and whether pericytes play an active role in the pathogenesis of these lesions.

## Mural cell heterogeneity: pericytes and smooth muscle cells and heterogeneity therein

Mural cell morphologies, molecular identities, and functional roles vary from one tissue to another, thereby complicating our ability to assign collective signatures to these cells. For example, distinct morphological features not only distinguish SMCs from pericytes, but also distinguish arterial SMCs from venous SMCs. Among microvascular mural cells, cell morphology changes along the AV connection (capillary), with three subtypes currently recognized—transitional pericytes (also called SMC/pericyte hybrid cells), mesh pericytes, and thin-strand pericytes (also called mid-capillary pericytes) (Smyth et al., 2018; Dalkara, 2019) (Figure 3). These morphological subtypes likely reflect varying functional roles for mural cells. For example, transitional pericytes, like their neighboring SMCs, typically found enwrapping the arteriole vessel segment, are predicted to be contractile and thus influence AV connection diameter, blood flow, and blood routing (Dalkara, 2019; Uemura et al., 2020). Moving toward the capillary bed, mesh pericytes may also exert contractile function, though likely to a lesser degree than transitional pericytes. Anatomically, mesh pericyte coverage of microvessels includes “pericyte-free holes” where the mesh-like cells begin to form cytoplasmic processes. In the microvasculature, or capillary bed proper, thin-strand pericytes—arguably the most well-documented pericytes—extend long, spindly, enwrapping, cytoplasmic processes from the cell body (Dalkara, 2019; Uemura et al., 2020). Given their relative abundance on the microvasculature, much of the reported reciprocal communication between vascular endothelial cells and pericytes likely involves thin-strand pericytes.

Mural cell heterogeneity may also be defined by unique, yet overlapping, gene expression signatures among cell types (Figure 3). As described above, SMCs and pericyte subtypes likely express varying levels the cytoskeletal gene *α-SMA* (*Acta2*), for example, to produce thick *versus* thin α-SMA fibers. While these overlapping expression domains present a challenge to identifying and labelling each distinct mural cell type, the field has adapted a multi-marker approach in which at least two mural cell markers (e.g., PDGFRβ, CD13 (aminopeptidase A/N), desmin, NG2 (*Cspg4*), *n-XlacZ4* (transgenic), α-SMA, Transgelin, 3G5, SUR2 (*Abcc9*), Kir6.1, DLK1, T-box18, GLAST, endosialin, CD29, CD90, CD146) (Kelly-Goss et al., 2013) are commonly used during data analyses. Further complicating the study of CNS mural cells, 1) expression of mural cells markers is dynamic, changing over time and at different stages of life (e.g., developmental *versus* mature); 2) expression of select markers may be enriched in specific subcellular locations (e.g., cell body *versus* processes); 3) marker expression may differ among mural cells within the CNS (e.g., brain *versus* spinal cord *versus* retina); 4) marker expression may change in healthy *versus* diseased tissue (e.g., AVM) (Armulik et al., 2011). Current methodological advances, such as single-cell RNA sequencing and multiplexed single-cell *in situ* RNA profiling, may help assign unique molecular signatures to each mural cell subtype and may even help identify new mural cell classifications. With continued technical advances, it is likely that functional specificity may also be assigned to mural cell subtypes, further highlighting mural cell heterogeneity.

## Mural cell heterogeneity reflects CNS AVM heterogeneity

While heterogeneity among mural cells presents challenges toward parsing out mechanisms of CNS AVMs, so too does mural cell heterogeneity provide opportunities for deeper understanding of the complex mechanisms underlying the associated pathologies. One proposed mechanism of AVM formation involves altered AV identity of endothelial cells comprising abnormal AVM vessels. Because AVMs are characterized by direct artery-to-vein shunts, at the expense of healthy capillaries, one can speculate that altered identity of an abnormal vessel segment (e.g., an AV shunt becomes “arterialized,” at the expense of venous gene expression) may affect the associated mural cell subtype, perhaps influencing gene expression or cell morphology to resemble a different mural cell type that is typically associated with another vessel segment (e.g., mural cells associated with arteries/arterioles, at the expense of mural cells associated with veins/venules). Another possibility is that during CNS AVM formation, one mural cell subtype acquires the identity of another mural cell. For example, consider that a thin-strand pericyte on a healthy capillary changes its molecular, morphological, and functional properties into those of an ensheathing (transitional) pericyte. Such cellular transdifferentiation, which has been described in pericytes, could explain how thin-strand pericytes might be involved in CNS AVM pathogenesis, even though thin-strand pericytes are *absent* from select CNS AVMs. Lineage tracing experiments in mouse models of CNS AVMs could help answer such unresolved questions. Increasingly, genomic studies and RNA sequencing data (bulk and single cell) report diverse gene mutations and aberrant gene expression in CNS AVM tissue (Fish et al., 2020; Winkler et al., 2022). Precise and novel roles for and consequences to mural cells will undoubtedly emerge as the CNS AVM pathogenesis narrative expands.

## Mural cell abnormalities: Cause or consequence of AVMs?

An unanswered question in the field is whether mural cell abnormalities are causal, during the initiation and/or progression of AVMs, or whether mural cell changes are a consequence of existing AVM pathology. Consistently, data from both human brain AVMs and mouse models of AVMs show severe pericyte loss in AVM tissue. Further, data show correlations between brain pericyte loss and vascular instability (characterized by more frequently ruptured vessels and increased vessel permeability) and between pericyte loss and more severe hemorrhages (Tu et al., 2006; Winkler et al., 2018; Sun et al., 2022). However, without a temporal experimental component, it is not possible to determine whether pericyte loss preceded or followed AVM pathologies. Thus, controlled, temporal studies are critical to testing among the possibilities of “cause” or “consequence.” Pathological time course data from mouse retina AVMs (Nadeem et al., 2020) shows that Rbpj-deficient mural cell loss from retina vessels precedes vessel enlargement. Similarly, deletion of *serum response factor* (*Srf*) from mural cells led to morphologically and functionally abnormal mural cells, followed by enlarged and leaky vessels that progressed to AV shunts (Orlich et al., 2022). These studies suggest that mural cell loss is required to permit abnormal vessel enlargement and AVM pathogenesis in the CNS. Conversely, some experimental AVMs, such as those following endothelial deletion of *Rbpj*, show increased pericyte area, which expands pathologically with increased endothelial area (Selhorst et al., 2022). Here, pericyte expansion more likely follows vessel enlargement, perhaps as part of a cellular response to maintain pericyte coverage of the abnormal vessels. Given the heterogeneity among AVMs, it is likely that different mechanisms lead to AVMs—some which require pericyte loss or other mural cells abnormalities for AVMs to proceed, and others which do not.

## Mural cell identity and function may be compromised during CNS AVM formation

In the CNS, mural cells are essential for maintaining vessel stability and barrier function, influencing blood flow and blood routing, and communicating with endothelial cells. What, then, does mural cell disturbance mean for CNS physiological health? To answer this query, the field must first piece together information about diverse mural cell identities and functions, then determine how mural cell perturbations are integrated during AVM onset, progression, and maintenance.

### Mural cell identity

As we described here, CNS AVMs are often characterized by loss or gain of mural cells or mural cell coverage (in many cases, data are specific for pericytes) associated with abnormal vessels. Where do pericytes go, or where do pericytes come from? Increased apoptosis accounts for reduced pericyte number and coverage following pericyte deletion of *Rbpj* (Nadeem et al., 2020). Transdifferentiation of pericytes, which thus lose their pericyte-specific molecular identity, may also account for pericyte loss. In support of this possibility, recent studies suggest reciprocal transdifferentiation between pericytes and mesenchymal stem cells (Tian et al., 2017; Yamazaki and Mukouyama, 2018). Further, evidence for transition of endothelial cells into mesenchymal cells (EndoMT) has been shown in human AVM tissue, suggesting that endothelial cells may be a course for mural cells via a two-step transformation (Shoemaker et al., 2020). Consistent with this reasoning, the previously mentioned fibromyocytes—which represent a potentially new mural cell cohort—show low expression of genes encoding contractile proteins, thus giving these cells an SMC-like identity (Winkler et al., 2022). Conversely, fibromyocytes do not express myocardin, a master regulator SMC molecular identity (Wang et al., 2003), thus distinguishing fibromyocytes from SMCs. Cell lineage tracing experiments will help researchers understand whether fibromyocytes indeed derive from a SMC lineage.

### Mural cells and the two-hit hypothesis

Evidence from human genomics and mouse genetics studies primarily shows that gene mutations (either germline or somatic gene mutations) in endothelial cells are the root cause of CNS AVM pathogenesis. However, data also suggest that other cells, including mural cells, perivascular astrocytes, and inflammatory cells (monocytes), are associated with AVM pathogenesis (Raabe et al., 2012; Zhang et al., 2016; Nadeem et al., 2020; Thomas et al., 2021; Orlich et al., 2022). For example, HHT-related AVMs are predicted to develop via a “two-hit” pathogenetic insult. Following this “two-hit” hypothesis, underlying genetic lesions are thought to contribute to the first pathological hit, while a later second hit to vessel stability places the vessel at risk for AVM formation. Perturbations to mural cells, either via an angiogenic insult or a direct cellular insult, may contribute to the second hit.

### Mural cells and blood flow

AVMs are characterized, in part, by abnormally high blood flow through AV shunts. The altered hemodynamics are predicted to affect endothelial cells directly, in response to increased blood flow and shear stress (Carlson et al., 2005; Murphy et al., 2014; 2012; 2008; Zhang et al., 2020). In turn, mural cell coverage of microvessels may increase, perhaps in response to a mechanical cue (vessel wall stretch) that promotes mural cell differentiation and maturation (Van Gieson et al., 2003). Indirect effects on mural cells, such as impaired recruitment to abnormal AVM blood vessels, have also been reported in an endothelial *Alk1*-deficient (Baeyens et al., 2016) mouse model of HHT. In two retinal HHT models [endothelial deletion of *Eng* (Mahmoud et al., 2010) or endothelial deletion of *Smad4* (Ola et al., 2018)], increased blood flow through AVM reprograms venous endothelial cells to acquire an arterialized identity and mural cells to upregulate α-SMA expression, resulting in impaired SMC organization along retinal vessels. Following mural cell deletion of *Srf* in mice, altered blood flow affects pericyte migration during vessel patterning, impairing angiogenic vessel integrity and tone (Orlich et al., 2022). In human patients, brain AVMs can be characterized as having high or low blood flow rates. Expression studies showed that SMCs from low flow rate brain AVMs had increased expression of WNT signaling mediators FZD10 and MYOC, compared to high flow rate SMCs (Huo et al., 2019), suggesting that activated WNT signaling may be involved in AVM formation. Collectively, these findings indicate that mural cells are indeed affected by the significant hemodynamic changes that accompany AVM pathologies.

## Consequences to mural cells following standard, clinical brain AVM treatment

The current, gold-standard treatment for AVM patients involves a team of medical specialists, social workers, physical therapists, and mental health providers, to treat symptoms and consequences of the disease. Without pharmacological treatments, clinicians use surgical methods—including radiation, embolization, resection, or some combination thereof—to treat CNS AVM in eligible patients (Figure 2). While surgical treatments may have great efficacy in reducing or resolving AVMs, it is not currently known how mural cells are affected. Radiation therapy, for example, delivers a focused dose of radiation to the AVM, thereby damaging cells of abnormal vessels and promoting vessel closure and shrinkage (Kano et al., 2017; Finitsis et al., 2020). However, irradiation also leaves weakened vessels prone to rupture—could changes to mural cells underlie the increased vessel fragility? While radiation therapy is an effective AVM treatment with potentially positive patient outcomes, little is known about accompanying damage to the peri-lesion brain tissue, including specific effects on mural cells. One possibility is that brain tissue injury induces inflammation, which in turn affects mural cells. In fact, mural cells may actively participate in the inflammatory response and/or tissue repair process. As mentioned above, pericytes have been shown to act as progenitor cells with stem-like properties and the potential for transdifferentiation. Activation of such programs in pericytes may promote lineages of cells involved in tissue repair and in response to inflammatory cues, such as those involved with fibrosis or astrogliosis. Clinical studies to analyze mural cell characteristics pre- and post-treatment will inform on possible cellular, molecular, and functional consequences to CNS mural cells.

## Therapeutics targeting mural cells in CNS AVMs

With increasingly understanding of mural cells’ involvement in AVM formation, these cells have become novel therapeutic targets for non-surgical, pharmacological treatments. Fundamentally, these approaches aim to increase vessel stability, to promote mural cell-microvessel interactions and mural cell attachment to microvessels, and to inhibit abnormal angiogenesis (Birbrair, 2019; Scherschinski et al., 2022).

The prevailing narrative toward targeting mural cells in AVMs stems from data administering the immunomodulatory drug thalidomide (or its derivative lenalidomide) either in pre-clinical models of AVM (Thalgott et al., 2015; Zhu et al., 2018) or in HHT patients to treat severe epistaxis (Lebrin et al., 2010; Balduini et al., 2012; Invernizzi et al., 2015). Thalidomide treatment in HHT patients restored endothelial PDGF-B expression, thus promoting recruitment of mural cells to blood vessels, strengthening pericyte attachment to microvessels, and enhancing vessel stability (Lebrin et al., 2010). Thalidomide administration in HHT mouse models yielded similar results. In *Eng*-deficient mice, thalidomide stimulated pericyte proliferation and increased PDGF-B expression in endothelial cells, thereby promoting pericyte recruitment and attachment to vessels (Zhu et al., 2018). In *Alk1*-deficient mice, thalidomide reduced vessel dysplasia and AVM bleeding and showed an anti-inflammatory response by reducing circulating inflammatory cells and inflammatory cytokines (Zhu et al., 2008; Zhu et al., 2018). While thalidomide treatment is a promising clinical therapy, it has surprisingly low specificity for mural cells, and it produces detrimental side effects. Analogs are under development, including pomalidomide, which is currently under clinical trial for HHT patients with severe epistaxis (https://clinicaltrials.gov/ct2/show/NCT03910244).

Other signaling pathways implicated in brain AVM may be targeted to attenuate mural cell abnormalities in AVMs. For example, a potential treatment is to target angiopoietins, which are reported to be upregulated in 30% of human brain AVMs (Scholz et al., 2015). Angiopoietins regulate mural cell migration, presumably away from AVM vessels, and thus are hypothesized to promote mural cell detachment. Treatments that increase PDGF-B/PDGFRβ signaling can presumably strengthen communication and interactions between endothelial cells and pericytes, to stabilize microvessels. As mechanisms of mural cell involvement in AVM continue to be uncovered, novel prevention and treatment strategies can be designed to test in animal models and to translate into effective therapies for AVM patients.

## Therapeutics targeting mural cells in other neurovascular/neurodegenerative diseases: future directions for CNS AVM therapies

Other neurovascular diseases (non-AVM), whose pathologies are characterized, at least in part, by perturbations to mural cells, have successfully manipulated mural cells in various therapeutic strategies. In a rat model of stroke (via middle cerebral artery occlusion), activation of miR-149-5p in pericytes increased N-cadherin expression and decreased microvessel permeability (Wan et al., 2018). Targeting PDGF-B/PDGFRβ signaling has been effective in models of neurovascular/neurodegenerative diseases. In a hypoxic cell culture model, mimicking ischemic stroke conditions, exogenous administration of PDGF-BB or TGFβ maintained microvessel integrity (Shen et al., 2019). Intravenous administration of PDGF-BB to epileptic mice reduced blood vessel leakage and normalized blood flow (Arango-Lievano et al., 2018). In an animal model of Parkinson’s Disease, PDGF-BB promoted neurovascular function via PDGFRβ signaling (Padel et al., 2016). This treatment was extended to human patients, as recombinant PDGF-BB was administered to Parkinson’s Disease patients, in a clinical trial to test the therapy’s safety (Paul et al., 2015). Finally, administration of the PI3K inhibitor cilostazol or the vasodilator iloprost (prostacyclin analog) shows promise in targeting mural cells and preserving vessel stability during stroke. Administration of either cilostazol or iloprost raised cAMP levels in pericytes and prevented pericyte detachment from endothelial cells, during stroke-inducing conditions in rats (Omote et al., 2014). These therapeutic agents also prevented pericyte loss and demyelination, following lysophosphatidylcholine (LPC) mediated disruption, thus maintaining vascular barrier function and reducing neuronal damage (Muramatsu et al., 2015). Both cilostazol and iloprost preserved blood-brain barrier function during oxygen/glucose deprivation, by inhibiting TGFβ signaling and strengthening tight junctions between endothelial cells (Takeshita et al., 2014).

Understanding mural cells in AVMs may inspire mechanistic insight and treatment options for other neurovascular diseases. In the inherited disease CADASIL (cerebral autosomal dominant arteriopathy with subcortical infarcts and leukoencephalopathy), mutations in *NOTCH3* lead to SMC degeneration or pericyte insufficiency, associated with blood vessel thickening and blood flow blockage (Joutel et al., 1996; Arboleda-Velasquez et al., 2011; Ghosh et al., 2015; Arango-Lievano et al., 2018). Like CNS AVMs, there are no pharmacological treatments currently available for CADASIL patients. Insight gained through the study of mural cells in one neurovascular disease may inform on pathogenic mechanisms and therapies for another.

## Conclusion

An experimental charge was put forth by Berthiaume et al. (Berthiaume et al., 2018) to identify anatomical zones, along a vessel, with distinct populations of mural cells, to assess gene expression in those populations, and to determine the various functions performed by those populations—functions that contribute to cellular contractility and regulation of blood flow, to vessel stability and permeability, to phagocytic removal of debris, and to service as stem cells and as a source of trophic signals. Progress made toward this charge is beginning to allow researchers to identify and study consequences to multiple different mural cell populations in AVMs. Continued studies will be aided by labeling and tracking mural cells *in vivo*, by *ex vivo* explant systems that manipulate and monitor intercellular interactions, and by continued genomics data. These future experimental goals will help us understand what mural cell heterogeneity means for initiation, progression, diagnosis, and treatment of CNS AVMs.
